# Impact of normalization and filtering on linkage analysis of gene expression data

**DOI:** 10.1186/1753-6561-1-s1-s150

**Published:** 2007-12-18

**Authors:** Joseph Beyene, Pingzhao Hu, Elena Parkhomenko, David Tritchler

**Affiliations:** 1 Department of Public Health Sciences, University of Toronto, Health Sciences Building 155 College Street, Toronto, Ontario, M5T 3M7, Canada; 2 Program in Population Health Sciences, The Hospital for Sick Children Research Institute, 555 University Avenue, Toronto, Ontario, M5G 1X8, Canada; 3 Program in Genetics and Genomic Biology, The Hospital for Sick Children Research Institute, 15-706 TMDT, 101 College Street, Toronto, Ontario, M5G 1L7, Canada; 4 Division of Epidemiology and Statistics, Ontario Cancer Institute, 610 University Avenue, Toronto, Ontario M5G 2M9, Canada

## Abstract

Using the Problem 1 data set made available for Genetic Analysis Workshop 15, we assessed sensitivity of linkage results to a correlation-based feature extraction method as well as to different normalization procedures applied to the raw Affymetrix gene expression microarray data. The impact of these procedures on heritability estimates and on expression quantitative trait loci are investigated. The filtering algorithm we propose in this paper ranks genes based on the total absolute correlation of each gene with all other genes on the array and has the potential to extract features that may play role in functional pathways and gene networks. Our results showed that the normalization and filtering algorithms can have a profound influence on genetic analysis of gene expression data.

## Background

Several recent studies have applied traditional quantitative trait linkage analysis to genome-wide gene expression data and have investigated the role of genetic variation in transcription [1]. These types of studies have the potential to uncover complicated transcriptional control. For example, Morley et al. and others identified several *cis*- and *trans*-acting genes and master regulators using linkage mapping on the gene expression phenotypes [1]. However, gene expression measurements are not generated in a uniform platform and, depending on the particular technology, most high-level statistical analyses using these data are preceded by a number of low-level pre-processing steps. For instance, Affymetrix GeneChip arrays have become a widely used microarray platform [2] and there are various algorithms for performing feature extraction and normalization on these high-density oligonucleotide gene expression arrays [3,4]. Ideally, all of the methods and algorithms should produce similar results. In practice, however, findings could be sensitive to variations in pre-processing approaches and may lead to different results. In particular, the impact that different algorithms have on expression quantitative trait loci (eQTL) analysis is not well understood. Understanding consequences of low-level pre-processing approaches is essential in interpreting findings from genome-wide linkage analyses of multivariate gene expression measurements. In this paper, we first propose a correlation-based filtering method. The motivation is to select genes that may participate in pathways. We then apply three normalization methods and the filtering algorithm to the Problem 1 data set made available for Genetic Analysis Workshop 15 (GAW15). In particular, we compare and contrast heritability estimates, concordance in top-ranked genes, and impact on the number of genes identified as *cis*-, *trans*- or multiple-regulators.

## Materials and methods

Raw gene expression measurements obtained from lymphoblastoid cell lines from 14 Centre d'Etude du Polymorphisme Humain (CEPH) Utah families were made available for GAW15. Furthermore, a subset consisting of 3554 genes that Morley et al. analyzed for linkage was also made available [1]. The genotypes of 2882 autosomal and X-linked SNPs of members of these families were generated by The SNP Consortium .

For the data in this problem, the Affymetrix Genome Focus Arrays were used. A unique feature of microarrays generated using the Affymetrix platform is the so-called MisMatch (MM) probes. Each of the probe pairs in a probe set has a Perfect Match (PM) and a MisMatch probe, with each probe having 25 bases. The PM probes are designed to bind perfectly to the gene of interest and the MM probes have a contrasting base at position 13 with the intention of measuring non-specific binding [2]. The Microarray Analysis Suite (MAS) algorithm from Affymetrix [2] incorporates information from the MM probes into the calculation of gene expression intensities. However, there is a continuing debate in the literature over the merit of MM probes as well as the impact of the various algorithms on downstream analysis. Several new algorithms have been proposed in recent years and most of them use only the PM signals to calculate gene expression. The algorithms include the robust multi-array average (RMA) method [3], Gene Chip RMA (GCRMA) [4], and the probe logarithmic error intensity estimate (PLIER) method from Affymetrix [2].

Morley et al. applied the MAS algorithm and selected 3554 genes using data from 94 grandparents such that the between-individual variance is larger than the within-individual variance [1]. In this report, we used raw CEL files from 194 subjects and applied the RMA, GCRMA, and PLIER algorithms to normalize and calculate gene expression intensities. Like the MAS algorithm, the PLIER method also incorporates information from MM probes while both RMA and GCRMA use only PM signals. We then ranked genes by the total absolute correlation (TAC) that each gene *i *has with the other *n *- 1 genes. The TAC for each gene is calculated as follows:

$$TAC_{i}=\sum_{j}^{n}\left|r_{ij}\right|-1,$$

where *r*_*ij *_is the correlation of gene *i *with gene *j*. For this filtering procedure, we used expression profiling data from 56 unrelated individuals. We extracted the top 3554 genes ranked by TAC from the largest ones to the smallest ones, so that we can make qualitative comparisons with what was reported by Morley et al. [1].

After the normalization and filtering steps, we carried out multipoint linkage analysis on each of the phenotypes using the MERLIN-REGRESS command in the statistical genetics software MERLIN [5]. The variance components (VC) option in MERLIN was used to estimate heritability. As in Morley et al., *cis *regulators were defined as those regulatory variants that mapped within 5 megabases (Mb) of the target gene [1]. All other significant linkages are categorized as *trans *regulators. Physical locations of probe sets were obtained from the Affymetrix annotation table . The Rutgers map was used to establish a correspondence between the physical map and the genetic map . Markers that could not be mapped using the Rutgers map, but that were located between physically anchored markers, were placed on the genetic map by interpolation.

## Results

It appears that the normalization methods have a strong effect on the gene expression correlation structure. The selected genes from RMA may have lower correlation than those from GCRMA and PLIER. Depending on the method used, different genes were identified as the top-ranking genes. The overlap in probe sets extracted using the three normalization approaches described earlier (RMA, GCRMA, and PLIER) along with our correlation-based feature extraction algorithm in comparison with the probe sets selected by Morley et al. [1] following their use of the Affymetrix MAS algorithm and a variance-based filtering varied from 10% to 43%. Specifically, the overlap between their results and the results from RMA, GCRMA, and PLIER were 1174/3554 (33%), 361/3554 (10%), and 1528/3554 (43%), respectively. Among these three methods, the rates of concordance were as follows: RMA versus GCRMA, 1670/3554 (47%); RMA versus PLIER, 1732/3554 (49%); and GCRMA versus PLIER, 1149/3554 (32%).

Table 1 presents a summary of the heritability estimates corresponding to the different normalization procedures. As can be seen from this table, the PLIER method resulted in a heritability estimate of less than 0.20 for all of the 3554 traits. Heritability estimates from data processed with RMA appear to be larger than the other two methods.

**Table 1:** Estimated heritability values based on the raw gene expression data sets normalized by three different methods

Heritability interval	RMA	GCRMA	PLIER
0.00 – 0.10	1592	3249	3510
0.11 – 0.20	863	145	44
0.21 – 0.30	609	82	0
0.31 – 0.40	309	42	0
0.41 – 0.50	134	23	0
0.51 – 0.60	29	10	0
0.61 – 0.70	13	2	0
0.71 – 0.80	4	1	0
0.81 – 0.90	1	0	0
0.91 – 1.00	0	0	0

Table 2 provides the number of expression phenotypes having *cis*-/*trans*-/*cis *and *trans*-regulators corresponding to the three normalization techniques and two *p*-value thresholds. The two *p*-value thresholds were chosen following Morley et al. for comparison purposes [1]. We have not identified *cis*-acting regulators among the top 3554 genes when using any of the three normalization methods and *p*-value thresholds. Few genes were regulated by both *cis*-and-*trans *SNPs. The number of expression phenotypes with a *trans*-regulator using RMA is comparable with results reported in Morley et al. using a *p*-value threshold of 3.7*10^-5^[1], in which they identified a total of 984 expression phenotypes with regulators. However, the GCRMA resulted in much fewer genes with *trans*-regulators while the PLIER method detected more genes with *trans*-regulators at the same *p*-value threshold.

**Table 2:** Number of expression phenotypes having *cis-/trans-/cis *and *trans*-regulators at different *p*-value thresholds using data sets normalized by three methods (*cis *regulators are those that mapped within 5 Mb of the target gene)

		RMA			GCRMA			PLIER		
				
*p*-Value	LOD	*cis*	*trans*	*cis*+*trans*	*cis*	*trans*	*cis*+*trans*	*cis*	*trans*	*cis*+*trans*
3.7*10^-5^	3.4	0	910	5	0	205	1	0	2527	2
4.3*10^-7^	5.3	0	454	1	0	71	0	0	258	0

A total of 67 expression phenotypes with strong linkage (*p*-value ≤ 3.7*10^-5^) have been detected by all three methods. 19 of the 67 (28.4%) expression phenotypes with *trans*-regulators that have been identified in all the data sets normalized using the three different normalized methods have also been shown to exhibit copy number variation in healthy individuals [6]. This was determined by searching the copy number variation database available at . These 19 expression phenotypes are listed in Table 3. The genes showing copy number variation and significant linkage signals may be of interest for further biological investigation.

**Table 3:** Selected expression phenotypes^a ^with significant evidence for linkage (LOD score ≥ 3.4) from genome scans identified in all data sets normalized by three different methods

		LOD score			
			
Gene symbol	Chromosomal location	RMA	GCRMA	PLIER	*cis*/*trans*
*MCP*	1q32	4.19	4.12	5.29	*trans*
*YT521*	4q13.2	3.79	4.51	4.38	*trans*
*RNF11*	1pter-p22.1	4.21	3.45	4.57	*trans*
*RASSF3*	12q14.2	4.99	4.60	5.17	*trans*
*RAB6C*	2q31	6.32	6.12	4.46	*trans*
*FAM8A1*	6p22-p23	4.62	4.75	5.20	*trans*
*ZNF217*	20q13.2	4.36	4.75	4.76	*trans*
*CREB3*	9pter-p22.1	3.60	4.41	3.48	*trans*
*PRPSAP1*	17q24-q25	4.97	3.54	4.28	*trans*
*SPAG9*	17q21.33	5.09	3.90	4.19	*trans*
*SRPK2*	7q22-q31.1	3.52	5.13	3.89	*trans*
*LOC81558*	17q21.33	3.96	5.44	4.36	*trans*
*IRF2*	4q34.1-q35.1	4.28	3.48	3.61	*trans*
*BCL2*	18q21.33|18q21.3	4.41	5.86	4.78	*trans*
*PTPN22*	1p13.3-p13.1	3.76	4.25	4.61	*trans*
*SDCBP*	8q12	5.76	4.76	5.08	*trans*
*OSBP*	11q12-q13	4.21	4.46	3.71	*trans*
*PTPN1*	20q13.1-q13.2	5.22	5.12	3.77	*trans*
*RGS12*	4p16.3	5.90	4.68	4.17	*trans*

^a^Some regions of these genes also exhibit copy number variation in healthy individuals (see details in the text).

**Figure 1:**
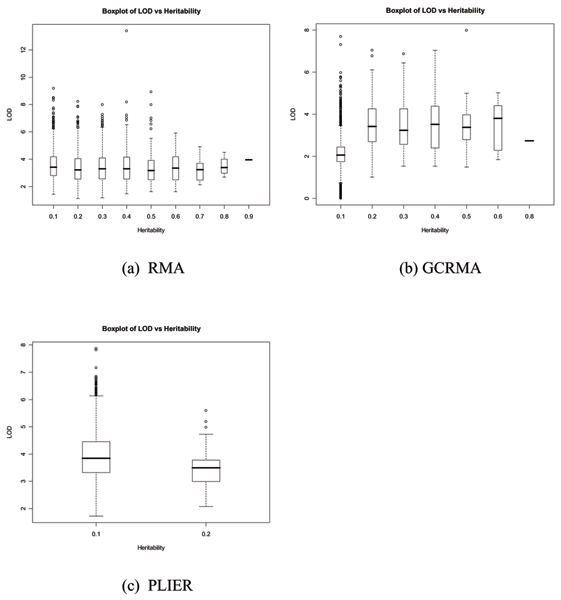
**Box plot of LOD scores versus heritability**. The plot shows the distributions of LOD scores at different heritability intervals for three normalization methods. For each gene, we took the maximum LOD score. a, RMA; b, GCRMA; c, PLIER.

We took the maximum LOD score for each trait and grouped them into different heritability categories. Figure 1 shows the overall trend of maximum values of LOD scores corresponding to different heritability intervals as shown in Table 1. No clear relationship between the heritability estimates and the maximum LOD score was seen. Some traits with high LOD scores have low heritability and other traits with high heritability have similar LOD scores as those with low heritability, as can be seen in Figure 1a and 1b.

## Discussion

Recently, a number of groups have started to integrate data from gene expression studies with genetic linkage analysis, leading to a new synergy between the two approaches [1]. Understanding the genetic basis of gene expression might shed light on processes that connect genotypic information to cellular- and organism-level information and systems biology. We used classical quantitative trait loci methodology in which expression levels are treated as quantitative phenotypes and genetic variants that significantly influence gene expression are sought along the entire genome. Several studies have shown that mRNA levels for many genes are heritable and mapping efforts have led to the characterization of genetic regulation in '*cis*', as well as in '*trans*' [1].

We applied three different normalization methods to the Problem 1 data set provided for GAW15 and carried out expression quantitative trait linkage (eQTL) analysis for 3554 traits. We used a filtering algorithm that extracts features based on a large total absolute correlation criterion to come up with the set of genes for the linkage analyses. Our findings suggest that different normalization and filtering algorithms can have a profound influence on genetic analysis of gene expression data. This observation is in agreement with a recent brief report by Williams et al. [7]. Our linkage analysis treated each trait independently of the others, similar to most other published work in this area [1]. Unlike the variance-based filtering used by Morley et al. [1], our correlation-based filtering takes dependence among genes into account in the pre-processing phase. The results from our approach can be useful in interpreting linkage results and inferring which genes may participate in pathways. We have not investigated whether the gene expression measurements are normally distributed, and this may also influence the power of the linkage findings.

Our choice of Pearson correlation was arbitrary but not critical. This is supported by the following analysis: we evaluated the correlations between the TAC estimated by Pearson (TAC-Pearson) and that by Spearman (TAC-Spearman) for the data normalized by the three normalization methods. The correlations between these two measures were 0.92, 0.90, and 0.94, respectively, for expression values normalized by RMA, GCRMA, and PLIER, respectively. We also evaluated the similarity of TAC rank of the set of 3554 expression phenotypes estimated by Pearson and Spearman correlations. For the 2000 top-ranked genes, the proportion of overlapped set of genes based on Pearson and Spearman were 89.8%, 87.2%, and 92.2% for RMA, GCRMA and PLIER, respectively.

## Competing interests

The author(s) declare that they have no competing interests.
